# Longer physical exercise duration prevents abnormal fasting plasma glucose occurrences in the third trimester: Findings from a cohort of women with gestational diabetes mellitus in Shanghai

**DOI:** 10.3389/fendo.2023.1054153

**Published:** 2023-01-24

**Authors:** Rui Zhang, Xiangjin Gao, Ting Sun, Huan Li, Qing Yang, Bin Li, Dongshan Zhu, Ruiping Wang

**Affiliations:** ^1^ Shanghai Skin Disease Hospital, Tongji University, Shanghai, China; ^2^ School of Public Health, Shanghai University of Traditional Chinese Medicine, Shanghai, China; ^3^ Obstetrics Department, Songjiang Maternal and Child Health Hospital, Shanghai, China; ^4^ School of Public Health, Shandong University, Jinan, Shandong, China

**Keywords:** gestational diabetes mellitus, abnormal plasma glucose, physical exercise duration, fasting plasma glucose, 2-h plasma glucose

## Abstract

**Objective:**

This study aims to investigate the relationship between daily physical exercise (PE) duration and frequency of abnormal plasma glucose (PG) times both during fasting and 2 h after a standard diet in women with gestational diabetes mellitus (GDM).

**Methods:**

We established a cohort involving 878 GDM women. GDM was confirmed by a diagnostic 75-g oral glucose tolerance test. Information was extracted from the delivery records and antenatal checkup forms. Physical exercise information was collected through a questionnaire.

**Results:**

Over 80% of GDM women were under 35 years old. An abnormal fasting PG with ≥1 occurrence presented in 742/878 (84.51%), and the abnormal PG 2 h after standard diet with ≥1 occurrence presented in 634/878 (72.21%). Compared to GDM women with ≥4 occurrences of abnormal fasting PG, GDM women with 0 occurrences (odds ratio (OR) = 2.56), one occurrence (OR = 1.94), two occurrences (OR = 2.29), and three occurrences (OR = 2.16) had a higher proportion of PE duration being in the 45–60-min/day group than those in the <45-min/day group, and GDM women also had a higher proportion of PE during being in the 61–90- and >90-min/day group than those in the <45-min/day group. However, the duration of PE was not associated to the number of abnormal PG occurrences 2-h after the standard diet.

**Conclusion:**

Moderate-intensity PE duration in GDM women was negatively associated with the number of abnormal fasting PG occurrences but not with the number of PG occurrences 2 h after the standard diet.

## Introduction

Gestational diabetes mellitus (GDM) is the abnormal glucose metabolism that occurs during pregnancy. In recent years, the diet and living habits of pregnant women have greatly changed with social and economic development, which has led to an obvious increase in the prevalence of GDM (1, 2). The International Diabetes Federation (IDF) estimated that approximately 14% of pregnancies and 18 million live births were affected by GDM worldwide in 2017 (3–5). In China, with the adoption of the universal second-child policy in October 2015 (6), more women in their 30s or 40s are planning to conceive. GDM prevalence in China continues to rise (7, 8), rising from 14.7% to 20.9% in recent years (9) due to advanced maternal age and a higher occurrence of pre-pregnancy overweight and obesity (10).

GDM is closely associated with impaired glucose tolerance and insulin resistance (1, 3), both of which have been linked to glucose, fat, and protein metabolism in pregnant women (11). GDM may cause serious complications during pregnancy (12, 13), including gestational hypertension, high infection risk, macrosomia, neonatal unit care admission, abortion, and premature birth (14). Thus, pregnant women with GDM are at high risk of having multiple adverse birth outcomes.

Maintaining the PG level in the normal range is key to reducing the occurrence of maternal and fetal complications (15). Nutritional control and physical exercise are basic interventions in the treatment and management of GDM (16). Growing evidence has demonstrated that physical exercises, including slow walking, swimming, stationary cycling, and yoga, are helpful in maintaining a normal PG level (17). The American College of Obstetricians and Gynecologists (ACOG) and the Canadian Society for Exercise Physiology (CSEP) issued an updated physical activity guideline in 2020, recommending at least 150 min of moderate-intensity physical activity per week for pregnant women without contraindications to achieve health benefits (18). However, Padayachee puts forward a different recommendation for GDM women considering the low-quality evidence in the ACOG- and CSEP-issued guideline (19). This specific guideline on physical exercise for GDM women recommended 30–60 min of moderate-intensity exercise three times per week (19). In a previous study, GDM women with more than 60 min of physical exercise presented a lower percentage risk of having abnormal plasma glucose (PG) levels (20, 21). However, evidence on the optimal amount and duration of physical exercise needed to curb the elevated glucose levels in GDM women as well as the effect of moderate-intensity physical exercise on glucose control during fasting and 2 h after diet remains limited.

In the women with GDM recruited for this study, we aimed to (1) estimate the prevalence of abnormal levels of PG tests during the third trimester in GDM women and (2) examine the effect of daily moderate-intensity physical exercise duration on the number of abnormal PG occurrences during the third trimester in women with GDM.

## Methods

### Participants

A prospective cohort of women with GDM was established in 2019 and 2020 in the Songjiang district of Shanghai, China. The purpose was to evaluate the impact of physical exercise on blood glucose control, preterm birth, adverse perinatal outcomes, and type 2 diabetes after delivery (21). GDM women at gestational weeks 24–28 were recruited from the Songjiang Maternal and Children’s Healthcare Hospital, Shanghai. The 75-g oral glucose tolerance test (OGTT) was used to diagnose GDM. The inclusion criteria were as follows: (1) aged 18–45 years; (2) gestational weeks 24–28; (3) confirmed GDM diagnosis with OGTT; (4) singleton pregnancy; (5) no preexisting health conditions such as diabetes, hypertension, or ischemic heart diseases; (6) currently living in Songjiang district of Shanghai; and (7) being able to read and sign an informed consent form (20, 21). GDM women with less than seven plasma glucose tests during routine antenatal checkups were excluded. The Review Board of Songjiang Maternal and Children’s Healthcare Hospital reviewed and approved this study (IRB#-2019-12-003). An informed consent paper was signed by each GDM woman before the questionnaire interview. This study was registered in the Chinese Clinical Trial Registry (ChiCTR2000028832) and was conducted in line with the Declaration of Helsinki.

### Data collection

Data were collected through a questionnaire that included three parts (20). Part A included five questions about the demographic features (age, education, monthly income, BMI before pregnancy, and residency status). Part B covered the pregnancy and childbirth history of GDM women, information on routine antenatal checkups, PG test records, and newborn delivery (gestational week, delivery mode, and information on any postpartum hemorrhage). Part C covered the performance of 20 types of physical activities during pregnancy (house cleaning, stationary bike riding, walking, jogging, swimming, climbing stairs, Tai Chi, soft gymnastics, yoga, etc.). The frequency and duration of each physical activity of moderate intensity undertaken during gestational weeks 27–40 were collected. Parts A and B were extracted from the hospital records. Part C of the questionnaire was collected by trained nurses through face-to-face interviews after the birth, while the mother was still in the hospital.

### Definitions of glycemic control status, physical exercise duration, and covariate

In this study, GDM was confirmed by a diagnostic 75-g OGTT at the gestational week of 24–28 when any one of the following values was met or exceeded (21): plasma glucose in fasting status (0 h) at ≥5.10 mmol/L, 1-h plasma glucose at ≥10.00 mmol/L, and 2-h plasma glucose at ≥8.50 mmol/L. Glycemic control status in the third trimester was indicated by the number of abnormal plasma glucose (PG) occurrences, which were tested in gestational weeks 27–28, 29–30, 31–32, 33–34, 35–36, 37–38, and 39–40. An abnormal PG in each antenatal checkup was defined as the fasting plasma glucose (FPG) ≥5.30 mmol/L or (and) postprandial blood glucose 2 h after a standard breakfast (PBG) ≥6.7 mmol/L, and the number of abnormal PG was counted both in total (FPG or/and PBG) and in separate forms (FPG or PBG). We classified GDM women into five different groups according to the occurrences of abnormal PG (groups with 0, 1, 2, 3, and ≥4 occurrences), and a higher number of abnormal PG occurrences indicates worse glycemic control. Daily moderate-intensity physical exercise duration was calculated using total daily activity times and the total weekly activity times (21). First, physical activity duration was calculated as monthly activity frequency multiplied by the average duration of each activity, then divided by 30 days. Thereafter, we combined the 20 types of physical activity duration to provide the total daily physical activity duration with moderate intensity for each participant. The daily physical exercise duration with moderate intensity was then divided into <45, 45–60, 61–90, and >90 min according to percentiles 25, 50, and 75. In addition, we classified women according to age: less than 35 years and 35 years or more. Education was recorded as completed years of schooling and categorized as 6–9 years (primary or junior high school), 10–12 years (senior high school), and >12 years (college and above) (20, 21). Individual monthly income was classified into three groups (<5,000 CNY, 5,000–10,000 CNY, and >10,000 CNY). BMI before pregnancy was divided into 14.5–23.9 kg/m^2^ (low or normal) and ≥24 kg/m^2^ (overweight, obesity) (20).

### Data analysis

Quantitative data are presented as means and standard deviations (SD) or median and interquartile range (IQR) as appropriate. We applied Student’s *t*-test or Mann–Whitney *U* tests to examine the difference between quantitative variables with a normal or skewed distribution. Categorical variables were described as frequency and percentage (%). The Chi-square test was used to test the difference in abnormal PG occurrences in GDM women with different demographic features.

Generalized linear regression (GLM) models were used to calculate the odds ratio (OR) and 95% confidence interval (95% CI) between physical exercise duration and the number of abnormal FPG or PBG occurrences, with the adjustment of potential confounders identified by directed acyclic graphs (DAGs) (22). For the GLM models, the reference groups were moderate-intensity physical exercise for <45 min, GDM women <35 years old, and abnormal occurrences of FPG (≥4 times in **Table 1**, >2 times in **Table 2**); or PBG ≥1 time (**Table 2**). SAS 9.4 (SAS Institute Inc.) was applied for analysis. A significant *p*-value was set at 0.05 or lower.

**Table 1:** The association between daily physical exercise duration and the number of abnormal fasting plasma glucose (FPG) occurrences in women with GDM (*n* = 878).

Duration of moderate-intensity physical exercise	Abnormal times of fasting PG	*n* (%)	Unadjusted OR and 95% CI	Adjusted OR and 95% CI
45–60 min/day	0 time	20 (11.76)	2.56 (1.21, 5.41)	2.48 (1.14, 5.38)
	1 time	19 (11.18)	1.97 (0.96, 4.05)	2.01 (0.96, 4.17)
	2 times	22 (12.94)	2.29 (1.14, 4.60)	2.30 (1.13, 4.70)
	3 times	26 (15.29)	2.16 (1.14, 4.11)	2.05 (1.07, 3.94)
	≥4 times	83 (48.82)	Ref	Ref
61–90 min/day	0 time	57 (17.92)	5.66 (2.94, 10.87)	6.33 (3.21, 12.46)
	1 time	59 (18.55)	4.76 (2.59, 8.73)	5.08 (2.72, 9.46)
	2 times	55 (17.30)	4.43 (2.41, 8.17)	4.83 (2.58, 9.02)
	3 times	40 (12.58)	2.58 (1.43, 4.67)	2.66 (1.46, 4.85)
	≥4 times	107 (33.65)	Ref	Ref
>90 min/day	0 time	46 (24.6)	13.22 (6.46, 26.97)	13.69 (6.5, 28.85)
	1 time	41 (21.93)	9.56 (4.83, 18.91)	10.05 (4.98, 20.26)
	2 times	35 (18.72)	8.16 (4.08, 16.33)	8.44 (4.14, 17.20)
	3 times	28 (14.97)	5.22 (2.65, 10.3)	5.39 (2.71, 10.72)
	≥4 times	37 (19.79)	Ref	Ref

Moderate-intensity physical exercise times at <45 min/group was taken as a reference for the polytomous explanatory variable of physical activity, and the number (%) of times of abnormal FPG in this group is 13 (6.40%) for 0 times, 16 (7.88%) for one time, 16 (7.88%) for two times, 20 (9.85%) for three times, 138 (67.98) for four times, and over. The age and times of production were adjusted in the GLM for the adjusted OR and 95% CI calculation.

PG, plasma glucose; OR, odds ratio; CI, confidence interval.

**Table 2:** The association between daily physical exercise duration and the number of abnormal FPG occurrences in women with GDM by age groups (*n* = 878).

Moderate-intensity physical exercise times	Abnormal times of fasting PG	Age <35 years		Age ≥35 years	
		*n* (%)	OR, 95% CI	*n* (%)	OR, 95% CI
45–60 min/day	≤2 times	34 (26.15)	1.8 (0.99, 3.28)	7 (35.00)	2.36 (0.6, 9.23)
	>2 times	96 (73.85)	Ref	13 (65.00)	Ref
61–90 min/day	≤2 times	94 (44.98)	4.17 (2.47, 7.05)	20 (38.46)	4.45 (1.43, 13.91)
	>2 times	115 (55.02)	Ref	32 (61.54)	Ref
>90 min/day	≤2 times	60 (57.69)	6.78 (3.71, 12.37)	16 (43.24)	4.13 (1.28, 13.29)
	>2 times	44 (42.31)	Ref	21 (56.76)	Ref

Moderate-intensity physical exercise times at <45 min/group was taken as a reference for the polytomous explanatory variable of physical activity. The number (%) of times of abnormal FPG in this group is 25 (16.13%) for ≤2 times and 130 (83.87%) for >2 times in GDM women less than 35 years and 7 (20.00%) for ≤2 times and 28 (80.00%) for >2 times in GDM women ≥35 years.

FPG, plasma glucose in fasting; OR, odds ratio; CI, confidence interval.

## Results

In this study, 878 GDM women were included in the final analysis. The overall mean (SD) age was 30.53 (3.95) years, with 81.21% of them under 35 years old. Of GDM women, 61.85% had an education in college and above, and the majority of them (77.11%) had a monthly income of over 5,000 CNY. 64.24% of women had lower or normal BMI before pregnancy, and 35.88% of them were local residents. The median value for gestation times, production times, and the number of live births were 2, 1, and 1, respectively (**Table 3**).

**Table 3:** The demographic feature in women with gestational diabetes mellitus (GDM) with different physical exercise times in Shanghai, China (*n* = 878).

Variables	Total GDM women	Moderate-intensity physical exercise times per day (min)				
		<45 min (*n* = 203)	45–60 min (*n* = 170)	61–90 min (*n* = 318)		>90 min (*n* = 187)
Age (years; *n* (%))						
18–34	713 (81.21)	167 (23.42)	148 (20.76)	262 (36.75)		136 (19.07)
35–45	165 (18.79)	36 (21.82)	22 (13.33)	56 (33.94)		51 (30.91)
Education (*n* (%))						
Primary/junior high	173 (19.70)	51 (29.48)	28 (16.18)	62 (35.84)		32 (18.50)
Senior high	162 (18.45)	33 (20.37)	38 (23.46)	59 (36.42)		32 (19.75)
College and above	543 (61.85)	119 (21.92)	104 (19.15)	197 (36.28)		123 (22.65)
Monthly income (CNY^a^; *n* (%))						
Less than 5,000 (US$<725)	201 (22.89)	52 (25.87)	39 (19.40)	76 (37.81)		34 (16.92)
5,000–10,000 (US$725–1,449)	456 (51.94)	104 (22.81)	98 (21.49)	158 (34.65)		96 (21.05)
Over 10,000 (US$>1,449)	221 (25.17)	47 (21.27)	33 (14.93)	84 (38.01)		57 (25.79)
BMI before pregnancy (*n* (%))						
14.5–23.9 (lower or normal)	564 (64.24)	117 (20.74)	112 (19.86)	203 (35.99)		132 (23.40)
≥24 (overweight, obesity)	314 (35.76)	86 (27.39)	58 (18.47)	115 (36.62)		55 (17.52)
Residency status (*n* (%))						
Local resident	315 (35.88)	78 (24.76)	65 (20.63)	110 (34.92)		62 (19.68)
Nonlocal resident	563 (64.12)	125 (22.20)	105 (18.65)	208 (36.94)		125 (22.20)
Times of gestation (median (IQR))	2 (1, 3)	2 (1, 3)	2 (1, 3)	2 (1, 3)		2 (2, 3)
Times of production (median (IQR))	1 (1, 2)	1 (1, 2)	1 (1, 2)	1 (1, 2)		2 (1, 2)
Live births (median (IQR))	1 (1, 2)	1 (1, 2)	1 (1, 2)	1 (1, 2)		2 (1, 2)
Times of abnormal PG in fasting (*n* (%))						
0 time	136 (15.49)	13 (6.40)	20 (11.76)		57 (17.92)	46 (24.6)
1 time	135 (15.38)	16 (7.88)	19 (11.18)		59 (18.55)	41 (21.93)
2 times	128 (14.58)	16 (7.88)	22 (12.94)		55 (17.30)	35 (18.72)
3 times	114 (12.98)	20 (9.85)	26 (15.29)		40 (12.58)	28 (14.97)
≥4 times	365 (41.57)	138 (67.98)	83 (48.82)		107 (33.65)	37 (19.79)
Times of abnormal PBG (*n* (%))						
0 time	244 (27.79)	52 (25.62)	57 (33.53)		94 (29.56)	41 (21.93)
≥1 time	634 (72.21)	151 (74.38)	113 (66.47)		224 (70.44)	146 (78.07)

a The exchange rate was 7.9.

FPG, fasting plasma glucose; PBG, postprandial blood glucose 2 h after a standard breakfast; CNY, Chinese Yuan; BMI, body mass index; IQR, interquartile range.

### Physical exercise in the third trimester in GDM women

The median duration of daily physical exercise was 60 min (IQR: 45–90). The proportion of women with daily physical exercise durations of <45, 45–60, 61–90, and >90 min were 23.12%, 19.36%, 36.22%, and 21.30%, respectively. GDM women aged ≥35 years with an education level of college and above, a higher monthly income, and lower BMI before pregnancy tend to have a higher proportion of daily exercise time over 60 min (**Table 3**).

### Plasma glucose status in routine antenatal checkup in GDM women

**Figure 1:**
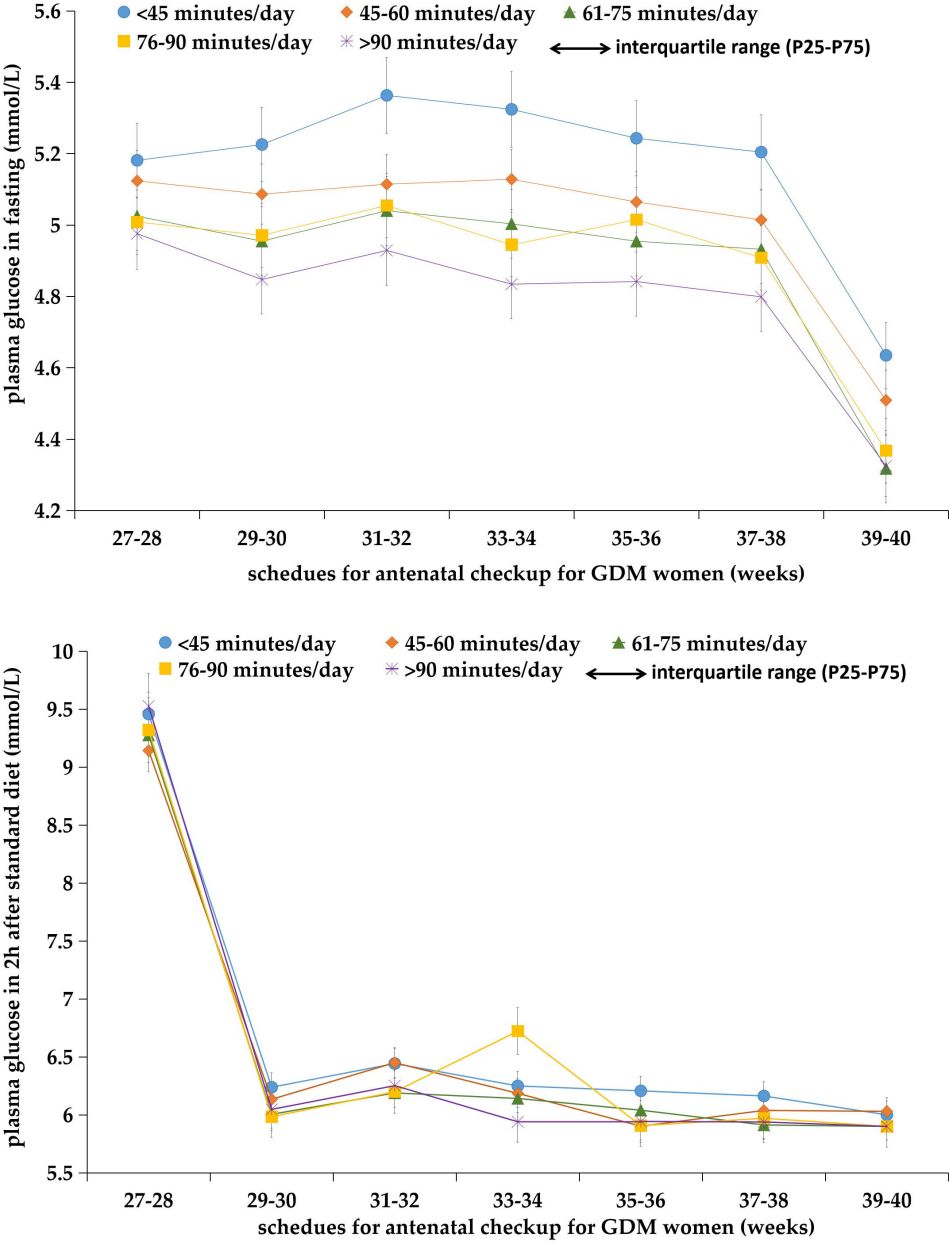
Plasma glucose levels both in fasting and 2 h after diet in GDM women with different levels of daily moderate-intensity physical exercise times at each scheduled antenatal checkup (*n* = 878).

In this study, 751 women with GDM had at least one abnormal PG test during the third trimester of pregnancy; the prevalence of total abnormal PG was 85.54%, with 84.51% (742/878) for FPG and 72.21% (634/878) for PBG. **Figure 1** shows the change in FPG and PBG levels in women with different physical exercise durations within each of the gestational weeks. GDM women with less daily physical exercise time (<45 min) tended to have higher FPG levels than those with more daily physical exercise time, but these differences were not obvious for PBG between GDM women with different levels of daily physical exercise time. The median abnormal PG occurrence was four times (IQR: 1–6) in all GDM women. The proportion of women with abnormal FPG of 0, 1, 2, 3, and ≥4 times were 15.49%, 15.38%, 14.58%, 12.98%, and 41.57%, respectively. GDM women aged 18–34 years old (16.13%) were more likely to have normal PG (i.e., 0-time abnormal occurrence) than women aged 35–45 years (12.73%). GDM women with a college or higher education (35.54%) and a monthly income of more than 10,000 CNY (33.94%) had a lower proportion of abnormal FPG at ≥4 times than those with a primary/junior high and senior high education or a monthly income of less than 10,000 CNY. GDM women with a BMI of ≥24 kg/m^2^ had a higher percentage of abnormal FPG at ≥4 times than those with BMI of <24 kg/m^2^ (**Table 4**).

**Table 4:** Abnormal fasting plasma glucose (FPG) times in gestational diabetes mellitus (GDM) women with seven routine antenatal checkups in Shanghai, China (*n* = 878).

Variables	Times of abnormal fasting PG in GDM women				
	0 time (*n* = 136)	1 time (*n* = 135)	2 times (*n* = 128)	3times (*n* = 114)	≥4 times (*n* = 365)
Age (years; *n* (%))					
18–34	115 (16.13)	107 (15.01)	106 (14.87)	91 (12.76)	294 (41.23)
35–45	21 (12.73)	28 (16.97)	22 (13.33)	23 (13.94)	71 (43.03)
Education (*n* (%))					
Primary/junior high	19 (10.98)	24 (13.87)	17 (9.83)	21 (12.14)	92 (53.18)
Senior high	13 (8.02)	22 (13.58)	20 (12.35)	27 (16.67)	80 (49.38)
College and above	104 (19.15)	89 (16.39)	91 (16.76)	66 (12.15)	193 (35.54)
Monthly income (CNY^a^; *n* (%))					
Less than 5,000 (US$<725)	20 (9.95)	29 (14.43)	28 (13.93)	28 (13.93)	96 (47.76)
5,000–10,000 (US$725–1,449)	75 (16.45)	62 (13.60)	61 (13.38)	64 (14.04)	194 (42.54)
Over 10,000 (US$>1,449)	41 (18.55)	44 (19.91)	39 (17.65)	22 (9.95)	75 (33.94)
BMI before pregnancy (*n* (%))					
14.5–23.9 (lower or normal)	112 (19.86)	97 (17.20)	85 (15.07)	75 (13.30)	195 (34.57)
≥24 (overweight, obesity)	24 (7.64)	38 (12.10)	43 (13.69)	39 (12.42)	170 (54.14)
Residency status (*n* (%))					
Local resident	56 (17.78)	51 (16.19)	48 (15.24)	40 (12.70)	120 (38.10)
Nonlocal resident	80 (14.21)	84 (14.92)	80 (14.21)	74 (13.14)	245 (43.52)
Times of gravidity (median (IQR))	2 (1, 3)	2 (1, 3)	2 (1, 3)	2 (1, 3)	2 (1, 3)
Times of parity (median (IQR))	1 (1, 2)	1 (1, 2)	1 (1, 2)	1 (1, 2)	2 (1, 2)
Live births (median (IQR))	1 (1, 2)	2 (1, 2)	1 (1, 2)	1.5 (1, 2)	2 (1, 2)

a The exchange rate was 7.9.

FPG, fasting plasma glucose; CNY, Chinese Yuan; BMI, body mass index; IQR, interquartile range.

### Association between physical exercise duration and number of abnormal FPG and PBG occurrences

In **Table 1**, we present the results of the multivariable analyses. Women with a longer physical exercise duration were more likely to have normal FPG tests (or fewer abnormal FPG occurrences) during the third trimester, compared to those with a daily physical exercise duration of less than 45 min. The odds of having 0-time abnormal FGP test were (OR: 2.56, 95% CI: 1.21–5.41), (OR: 5.66, 95% CI: 2.94–10.87), and (OR: 13.22, 95% CI: 6.46–26.97) for women with a daily physical exercise duration of 45–60, 61–90, and >90 min, respectively. When analyses were stratified by maternal age, in women with maternal ages less than 35 years, longer physical exercise duration was linked to fewer abnormal FPG tests (≤2 times), and there was a dose–response trend. Women with a physical exercise duration of 61–90 min had the highest odds of having less times of abnormal FPG tests (≤2 times) (OR: 4.45, 95% CI: 1.43–13.91) in women with a maternal age greater than 35 years (**Table 2**). Whereas, physical exercise duration was not associated with abnormal PBG occurrences tested 2 h after the standard diet (**Table 5**; **Figure 1**).

**Table 5:** The association between daily physical exercise duration and times of abnormal PBG in women with GDM.

Moderate-intensity physical exercise times	Abnormal times of PBG	*n* (%)	Unadjusted OR and 95% CI	Adjusted OR and 95% CI
45–60 min/day	0 time	57 (33.53)	1.47 (0.94, 2.29)	1.38 (0.88, 2.18)
	≥1 time	113 (66.47)	ref	ref
61–90 min/day	0 time	94 (29.56)	1.22 (0.82, 1.81)	1.21 (0.81, 1.81)
	≥1 time	224 (70.44)	ref	ref
>90 min/day	0 time	41 (21.93)	0.82 (0.51, 1.30)	0.82 (0.51, 1.32)
	≥1 time	146 (78.07)	ref	ref

Moderate-intensity physical exercise times at <45 min/group was taken as a reference for the polytomous explanatory variable of physical activity. The number (%) of times of abnormal PBG (plasma glucose 2 h after a standard breakfast) in this group is 52 (25.62%) for 0 times and 151 (74.38%) for one time or more. Age was adjusted in the GLM for the adjusted OR and 95% CI calculation.

PBG, postprandial blood glucose 2 h after a standard breakfast; OR, odds ratio; CI, confidence interval.

## Discussion

In our previous study based on the GDM women cohort, we noticed that GDM women with more exercise times had a lower percentage of abnormal PG, especially when exercise times were ≥60 min/day (20) and that women who are multiparous have less effective glycemic control through physical activity, so multiparas need more physical activity to achieve glycemic control at a similar level to primiparas (21). In this study, we selected 878 GDM women from the same cohort who had seven times plasma glucose tests during routine antenatal checkups to see if more daily physical exercise duration could prevent the abnormal fasting PG occurrences as well as the abnormal PG 2 h after standard diet in the third trimester in women with GDM. We identified that an abnormal PG in the third trimester was prevalent in women with GDM, with up to 42% of GDM women having an abnormal FPG of ≥4 times and 72% of GDM women having an abnormal PBG of ≥1 time. GDM women with longer moderate-intensity physical exercise times had a lower number of abnormal fasting PG occurrences. In contrast, the times of abnormal 2-h PG tests were not associated with physical exercise. The association between longer physical activity duration and a lower number of fasting abnormal PG occurrences in GDM women might be due to the fact that insulin resistance might be influenced or controlled by physical exercise in women with GDM. Insulin resistance can increase energy supply for lipid oxidation, promote glucose phosphorylation in muscle cells, and transform blood sugar to myosin to make glucose more stable, thereby maintaining the balance between glucose and insulin secretion (21).

In this study, we noticed that GDM women aged ≥35 years had a higher proportion of abnormal PG times of ≥4 times than those aged <35 years, even with more physical exercise times. This could be because GDM women aged ≥35 years were more likely to be multiparous women with more glycemic control problems (23). Previous studies indicate that the increasing recurrence of GDM is positively associated with the increasing number of births in women (24–26). One explanation for the possible mechanism is that these episodes of insulin resistance may contribute to the decline in β-cell function since each pregnancy is characterized by an episode of insulin resistance (21, 27). Furthermore, the influence of parity on glycemic control in GDM women may be due to previous pregnancies affecting insulin sensitivity and glucose metabolism; in addition, they were also slightly more overweight (21). Findings in this study provide evidence that GDM women of older age were susceptible to poor glycemic control, and they extend the research outcomes by showing that longer physical exercise duration is needed for GDM women to obtain a beneficial response.

Previous studies indicated that it was possible to control the PG through physical exercises (20). Previous research also reported that insufficient exercise was an independent risk factor for type 2 diabetes mellitus (T2DM) and was also an inducement of metabolic syndrome (28). The American College of Sports Medicine and the American Diabetes Association Joint Position Statement recommend that physical exercise is a key content in T2DM prevention and management and has physical and psychological benefits for GDM women (29). In this study, we noticed that the proportion of abnormal PG was lower in GDM women with longer physical exercise duration, both in GDM women aged <35 and ≥35 years, which indicates that the number of abnormal PG occurrences was negatively associated with physical exercise duration. This can be explained by the fact that physical exercise may actively affect insulin resistance in patients with GDM (21, 29). Insulin resistance can increase energy supply for lipid oxidation, promote glucose phosphorylation in muscle cells, and transform blood sugar to myosin to make glucose more stable, thereby maintaining the balance between glucose and insulin secretion (21). Moreover, studies have also validated that exercise can increase the utilization of glucose, improve blood glucose status, and reduce basic insulin resistance during pregnancy; it is one of the PG control measures for GDM (30, 31). On the other hand, more potential benefits of physical exercise for GDM women include improved fitness, less gestational weight gain, and reduced risk of hypertensive disorder (32, 33). In this study, we also noticed that physical exercise times in GDM women were not statistically correlated with the times of abnormal 2-h PG tests. This might be due to the standard diet with limited energy provided by the hospital, which makes the PG level more stable in the 2-h test, but the true cause should be explored in future studies.

Referencing the 2019 Canadian guidelines for physical exercise during pregnancy, which suggest moderate-intensity physical exercise of ≥150 min/week or moderate-intensity exercise for 30–60 min at a frequency of three times per week (18). In this study, the median value of daily physical exercise time was 60 min in women with GDM, which was higher than the ACOG recommendation. The higher daily physical exercise times in GDM women than recommended might be due to differences in physical exercise types in Chinese GDM women compared with those in western countries. In China, pregnancy is treated as a special state that requires extra rest and recuperation (20), so physical activities such as soft gymnastics, oxygen dance, and swimming are unacceptable to the majority of pregnant women in China. Whereas aerobic exercise with a lower intensity, which mainly refers to the continuous exercise participated by the large muscle groups of the body, is more acceptable in GDM women in China. Walking is a simple and commonly used aerobic exercise (34). Daily walking does not require special equipment and may be the most suitable physical activity for pregnant women (35). Moreover, resistance exercise, also known as strength training, can help avoid discomfort caused by fetal growth and a forward shift in body weight in late pregnancy and can be performed at home or even in bed, making it an additional option for GDM women (20). So, considering the fact that physical exercise provides benefits, physical activity of any type with proper intensity and duration can have benefits for GDM women.

This study is to elaborate on the association between the duration of moderate-intensity physical exercise and the number of abnormal PG in women with GDM in Shanghai, China. One strength of this present study is the cohort with a large population size, which included 878 GDM women with all seven PG tests. Meanwhile, information on demographic features and detailed information for each PG test were directly extracted from the routine antenatal checkup forms and the delivery records of GDM women in the hospital; this contributed to the relatively less measurement bias and recall bias, which is another strength of this current study.

This study has several limitations that should be taken into consideration. Firstly, the data analyzed in this study are extracted from a previously established GDM women cohort; the sample size is not sufficiently large for a detailed subgroup analysis. Secondly, this study only recruits women with GDM in Shanghai, which does not cover GDM women in other regions of China. It is arguable that there may be differences in lifestyle in GDM women in different cities, which limits the generalization of the findings in this study. Thirdly, data for each type of physical exercise duration in GDM women are collected by self-reported questionnaire interview, which might induce report bias for the daily physical exercise duration assessment. Fourthly, diet assessment in GDM women is not performed in this study, so we miss the chance to investigate and analyze the combined effect of diet and physical exercise on abnormal PG levels in GDM women, even though we assume that there is no difference in diet in GDM women with different physical exercise times. Therefore, the incorporation of some improvement should be considered in further follow-up studies.

## Conclusions

Abnormal PG occurrences in the third trimester were prevalent in women with GDM. Longer duration of moderate intensity physical exercise per day was associated with a fewer number of abnormal FPG occurrences, but not the number of PG occurrences 2 h after the standard diet. For GDM women with maternal age greater than 35 years, 45–60 min of daily physical exercise might be appropriate for glucose control.

## Data availability statement

The raw data supporting the conclusions of this article will be made available by the authors, without undue reservation.

## Ethics statement

The studies involving human participants were reviewed and approved by The ethics approval was approved by Songjiang Maternal and Childcare Hospital Institution Review Board (IRB#2019-12-003). An informed consent paper was signed by each participant before the questionnaire interview. The patients/participants provided their written informed consent to participate in this study.

## Author contributions

RPW, BL and DSZ participated in study design. RZ and XJG conducted the study and drafted the paper. TS, HL and QY participated in field work. RPW revised the paper, all authors have read this paper and approved the final manuscript. All authors contributed to the article and approved the submitted version.
